# Artificial intelligence in the diagnosis and treatment of acute appendicitis: a narrative review

**DOI:** 10.1007/s13304-024-01801-x

**Published:** 2024-03-12

**Authors:** Valentina Bianchi, Mauro Giambusso, Alessandra De Iacob, Maria Michela Chiarello, Giuseppe Brisinda

**Affiliations:** 1 Emergency Surgery and Trauma Center, Department of Abdominal and Endocrine Metabolic Medical and Surgical Sciences, IRCCS, Fondazione Policlinico Universitario A Gemelli, Largo Agostino Gemelli 8, 00168 Rome, Italy; 2 General Surgery Operative Unit, Vittorio Emanuele Hospital, 93012 Gela, Italy; 3 Department of Surgery, General Surgery Operative Unit, Azienda Sanitaria Provinciale Cosenza, 87100 Cosenza, Italy; 4 Catholic School of Medicine, University Department of Translational Medicine and Surgery, 00168 Rome, Italy

**Keywords:** Acute appendicitis, Artificial intelligence, Diagnosis, Laparoscopy, Surgery

## Abstract

Artificial intelligence is transforming healthcare. Artificial intelligence can improve patient care by analyzing large amounts of data to help make more informed decisions regarding treatments and enhance medical research through analyzing and interpreting data from clinical trials and research projects to identify subtle but meaningful trends beyond ordinary perception. Artificial intelligence refers to the simulation of human intelligence in computers, where systems of artificial intelligence can perform tasks that require human-like intelligence like speech recognition, visual perception, pattern-recognition, decision-making, and language processing. Artificial intelligence has several subdivisions, including machine learning, natural language processing, computer vision, and robotics. By automating specific routine tasks, artificial intelligence can improve healthcare efficiency. By leveraging machine learning algorithms, the systems of artificial intelligence can offer new opportunities for enhancing both the efficiency and effectiveness of surgical procedures, particularly regarding training of minimally invasive surgery. As artificial intelligence continues to advance, it is likely to play an increasingly significant role in the field of surgical learning. Physicians have assisted to a spreading role of artificial intelligence in the last decade. This involved different medical specialties such as ophthalmology, cardiology, urology, but also abdominal surgery. In addition to improvements in diagnosis, ascertainment of efficacy of treatment and autonomous actions, artificial intelligence has the potential to improve surgeons’ ability to better decide if acute surgery is indicated or not. The role of artificial intelligence in the emergency departments has also been investigated. We considered one of the most common condition the emergency surgeons have to face, acute appendicitis, to assess the state of the art of artificial intelligence in this frequent acute disease. The role of artificial intelligence in diagnosis and treatment of acute appendicitis will be discussed in this narrative review.

## Introduction

Artificial intelligence (AI) was born in the 1950s [1], spreading in various fields in the last years including statistics, psychology, linguistics, and also medicine and surgery [2–6]. Surgeons must take decision for a correct diagnosis and treatment during their daily activity. This is not always easy and AI could have a role in this scenario. In fact, the aim of AI in medicine is to solve real-life clinical problems, achieving a result that has to be similar or even better than human mind’s [7, 8]. Specific applications of AI in surgery are preoperative risk prediction, intraoperative video analysis and electronic health records. Physicians have assisted to a spreading role of AI in the last decade and this involved different medical specialties such as ophthalmology [9], cardiology [10], urology [11, 12], but also colorectal [13–15], gastric [16, 17], hepatobiliary and pancreatic surgery [18–21]. Its role in the emergency departments has also been investigated [22–26]. De Simone et al.[27, 28] conceived the Artificial Intelligence in Emergency and Trauma Surgery project aiming at increasing AI availability for emergency surgeons. They carried out a survey to assess the relationship between AI and the emergency surgeons. It came out that most of them has an interest in this field and believes that could be helpful in improving clinical outcomes and surgical education. We considered one of the most common condition the emergency surgeons have to face, acute appendicitis, to assess the state of the art of AI in this frequent acute disease. For this reason, the role of AI in diagnosis and treatment of acute appendicitis will be discussed in the next sections.

## Terminology of AI

AI has various subfields (Table 1). Machine learning (ML) [29] is certainly the most developed branch. It is based on algorithms which provide answers from a statistical analysis on large datasets. ML can be supervised, in which input objects and a desired output value train a model, or unsupervised [6]. Among the supervised ML techniques, one of the most widely used is logistic regression (LR). It is a statistical model used in machine learning classification algorithms to obtain the probability of belonging to a certain class. LR belongs to generalized linear models (GLM), a flexible generalization of ordinary linear regression.

**Table 1:** Terminology of artificial intelligence

Machine learning	Involves programming algorithms to make decision or predictions based on data
Deep learning	A type of machine learning that utilizes artificial neural networks for processing and analyzing a significant amount of data
Natural language processing	A branch of artificial intelligence that aims to teach computers how to comprehend and analyze human language
Robotics	The study and the development of robots capable of performing tasks autonomously or with human assistance
Computer vision	Machines’ ability to interpret and comprehend images and video
Expert systems	Systems of artificial intelligence that mimic a human expert’s decision-making abilities in a specific field
Neural networks	Computer systems that are designed to mimic the structure and function of the human brain
Cognitive computing	Systems of artificial intelligence that can simulate human thought processes, including perception, reasoning, and problem-solving
Natural language generation	The process of using artificial intelligence to generate human-like language in written or spoken form
Data mining	The revelation of patterns and insights in large datasets
Bayesian networks	A probabilistic graphical model used for probabilistic reasoning
Swarm intelligence	The collective behavior of decentralized, self-organized systems
Decision trees	A graphical representation of decision-making processes
Support vector machines	A machine learning algorithm used for classification and regression analysis
Human-in-the-loop	A type of system of artificial intelligence that incorporates human feedback to improve performance

Another widely used supervised model is random forest (RF) [30]. It is an ensemble learning method for classification, regression and other tasks that operates by constructing a multitude of decision trees at the time of training. RF aim is to achieve good predictive results through randomization of predictive factors. Support vector machine (SVM) [31], k-nearest neighbors (K-NN) [32], naïve bayes (NB) [33] are other frequently used supervised ML models. Decision tree (DT) [34] is a ML model that represents a series of logical decisions made on the basis of attribute values. DT consists of a tree structure that is used to make decisions or predictions from input data. This large family is represented, amongst many, by gradient boosted tree (GBT), classification and regression tree (CART), and even RF, which combine the simplicity of decision trees with the flexibility and power of an ensemble model. Unlike supervised learning, unsupervised learning does not utilize a prespecified annotation; rather, it draws inferences from unlabeled data to identify patterns and/or structure within a data set. This type of learning can be useful in identifying relationships between groups (e.g., clustering) for further hypothesis generation [35].

Deep learning (DL) is a more complex form of ML, able to learn features and to use them for diagnostic purposes [36, 37]. The adjective “deep” refers to the use of multiple layers in the network. This branch is based on artificial neural networks (ANN), models that are built using principles of neuronal organization discovered by connectionism in the biological neural networks constituting animal brains. Hence, they simulate the human brain and its neural connections. It is still underused in medicine because of its complexity [38].

## AI in diagnosis of acute appendicitis

Diagnosis of appendicitis can sometimes be challenging even for the most experienced surgeons. The latest guidelines of the World Society of Emergency Surgery on diagnosis and treatment of appendicitis [39] indicate that an integration of clinical, biochemical markers and imaging is necessary for the diagnosis of this condition, considering factors as the patient’s age, gender and comorbidities. Diagnostic tools such as the Alvarado score, the appendicitis inflammatory response and the adult appendicitis score are sensitive enough to suspect acute appendicitis. Laboratory markers as leukocytosis or elevated C-reactive protein are helpful for arising suspect of this pathology, especially for the complicated forms [39]. The gold standard for appendicitis radiologic diagnosis remains abdominal ultrasound, if performed by an experienced operator, both for adults and children. Second level investigations, preferably low-dose CT-scan, are to be considered in doubtful cases.

The use of AI in the diagnosis of acute appendicitis is still emerging [40–43]. A small number of studies have been published so far. The achievements of various research proposed in recent years in the field of ML and sophisticated human anatomy designed DL have been remarkable [43–45]. The aim of several studies was primarily the diagnosis of acute appendicitis, but also the differentiation between complicated and uncomplicated forms [40–42, 46–48]. AI training was based on data as demographics (gender and age), clinical (abdominal pain or other associated symptoms), biomarkers (especially leucocyte counts and C-reactive protein), and imaging techniques (abdominal ultrasound or CT-scan). In most cases, the input data were represented by a different combination of these factors.

Among ML models used, RF has proven to be the most accurate for acute appendicitis diagnosis. According to a prospective study by Aydin et al. [49], after adequate pre-training with demographic, clinical and laboratory data, RF showed the best results in terms of accuracy, sensitivity and specificity against the other ML methods analyzed (area under the cure—AUC 0.99). This result was not only for the diagnosis of acute appendicitis, but also for detecting the complicated forms. RF optimal results in diagnosing appendicitis were also observed by Hsieh et al. [50], with percentages of accuracy, sensitivity and specificity above 90%; input data were represented by a combination of demographics, clinical and biomarkers. Anyhow, these results have a limited significance because of the retrospective nature of the study and the number of case series. On the other hand, reduced sensitivity and specificity of RF technique was found by Mijwil et al. [51]; in this study AI training was performed exclusively with laboratory data. Nevertheless, DT models were preferred because of their greater ease of interpretation and simplicity of use in medical practice. Additionally, in a recent Brunei study [52] DT predictive model proved very useful in diagnosing acute appendicitis, with accuracy rates of 97%.

Other ML models have been investigated, too. Among them, the GBT, followed by RF, has been shown to be particularly effective in diagnosing appendicitis according to Akmese et al. [47]. Catboost, a supervised GBT on DT algorithm able to work with categorical data, found 92% accuracy in distinguishing perforated from non-perforated acute appendicitis [53]. The primary focus of some recent studies has been the identification of complicated appendicitis, through ML techniques that have proven to be particularly accurate such as RF, SVM [54] and GBT [55]. Reisman et al. [56], using bootstrapping resampling machine learning techniques, discriminated between phlegmonous and gangrenous forms of acute appendicitis on the basis of an extensive genetic analysis of 56,666 genes, revealing how gene expression may underlie the pathophysiology of these disease patterns. K-NN, DT, SVM, CART, NB, linear and LR [57] are other ML models that have been tested, with less promising results [45].

In the last few years, DL, a subset of AI based on ANN, has been increasingly used, with excellent results. ANN have proven to be particularly effective for the diagnosis of acute appendicitis [43, 52]. Prabhudesai et al. [58] showed that the use of ANN was better to exclude false appendicitis compared to the clinic and Alvarado score, significantly. It showed a sensitivity of 100% and specificity of about 97%. Yoldaş et al. [59] showed comparable results in identifying acute appendicitis. In 2015, Park et al. [60] worked on a case series of 801 patients after pre-training with Alvarado score. ANN demonstrated to have a high sensitivity and specificity in diagnosing acute appendicitis (close to 100%) compared to Alvarado score (*P* < 0.001). Recently, a subset of ANN, the convolutional neural networks (CNN) have been used in the processing of *AppendiXnet *[61]. This 18-layer 3D CNN is a form of AI able to diagnose acute appendicitis using a training dataset of 438 CT-scan exams after pretraining on a large collection of YouTube human videos called Kinetics. This pretraining was able to significantly improve the performance of the model in detecting appendicitis from an AUC of 0.72 to 0.81. The role of AI as an aid for the diagnosis of acute appendicitis (Table 2) seems to be promising, with DL techniques as ANN that seem to prove superior to more classical ML techniques [44, 46, 57, 61]. Further studies, with more conspicuous and multicenter data, would certainly help in the validation of this important diagnostic tool.

**Table 2:** A summary of the articles describing the role of artificial intelligence in the diagnosis of acute appendicitis

Author	Year	Country	Method	Aim	Findings
Aydin et al. [49]	2020	Turkey	n 7244 ML (**RF**, **DT**, K-NN, NB, SVM, GLM) Training: demographic data and laboratory markers	Ability to diagnose acute appendicitis and differentiate uncomplicated from complicated forms	RF best results (AUC 0.99) DT easier interpretability (AUC 0.94)
Shikha et al. [52]	2023	Brunei	n 166 ML (**DT**) Training: clinical and laboratory markers	Diagnosis of acute appendicitis	DT accuracy 97%
Hsieh et al. [50]	2011	Taiwan	n 180 ML (**RF**, SVM, LR), DL (ANN) Training: demographic and clinical data including Alvarado score	Diagnosis of acute appendicitis	RF best results (sensitivity 94%, specificity 100%, accuracy 96%, AUC 0.98)
Mijwil et al. [51]	2022	Iraq	n 625 ML (**RF**, NB, GLM, LR, DT, GBT, SVM) Training: laboratory markers	Diagnosis of acute appendicitis	RF best results (sensitivity 81%, specificity 81%, accuracy 84%)
Akmese et al. [47]	2020	Turkey	n 595 ML (**GBT**, RF, K-NN, LR, SVM, CART), DL (ANN) Training: demographic data and laboratory markers	Diagnosis of acute appendicitis	GBT best results (accuracy 95%)
Akbulut et al. [53]	2023	Turkey	n 1797 ML (**Catboost**) Training: demographic and biochemical data	Detect acute appendicitis and predict perforated and non-perforated appendicitis	Diagnosis of acute appendicitis (accuracy 88%, sensitivity 84%, specificity 93%, AUC 0.94) Detect perforated appendicitis (accuracy 92%, sensitivity 94%, specificity 90%, AUC 0.97)
Xia et al. [54]	2022	China	n 298 ML (**SVM**) Training: demographic and clinical data, biomarkers	Differentiate complicated from uncomplicated appendicitis	SVM accuracy 84%, sensitivity 82%, specificity 85%
Phan-Mai et al. [55]	2023	Vietnam	n 1950 ML (**GBT**, K-NN, DT, LR, SVM), DL (ANN) Training: demographic data, biomarkers and US	Differentiate complicated from uncomplicated appendicitis	GBT best results (AUC > 0.80)
Reismann et al. [57]	2021	Germany	n 29 ML (**Bootstrap**) Training: biomarkers (genes)	Detect phlegmonous and gangrenous appendicitis	Set of 4 genes as discriminatory biomarkers (AUC 0.84)
Ghareeb et al. [45]	2021	Egypt	n 319 ML (**K-NN**, DT, LR, NB, SVM) Training: Alvarado score, US and combined US-Alvarado score	Diagnosis of acute appendicitis	K-NN best results (accuracy 91%, AUC 0.82)
Reismann et al. [56]	2019	Germany	n 590 ML (**LR**) Training: laboratory markers and US	Ability to diagnose acute appendicitis and differentiate uncomplicated from complicated forms	Diagnosis of acute appendicitis (sensitivity 93%, specificity 67%, accuracy 90%) Identifying complicated appendicitis (sensitivity 95%, specificity 33%, accuracy 51%)
Prabhudesai et al. [58]	2008	UK	n 60 DL (**ANN**) Training: Alvarado score and clinical assessment	Diagnosis of acute appendicitis	ANN sensitivity 100%, specificity 97%
Yoldaş et al. [59]	2012	Turkey	n 156 patients DL (**ANN**) Training: clinical assessment and laboratory markers	Diagnosis of acute appendicitis	ANN sensitivity 100%, specificity 97%, AUC 0.95
Park et al. [60]	2015	Korea	n 801 DL (**ANN**) Training: Alvarado score	Diagnosis of acute appendicitis	ANN sensitivity 99–100%, specificity 96–99%
Rajpurkar et al. [61]	2020	USA	n 438 DL (**CNN**) Training: CT-scans after *Kinetics* YouTube video pre-training)	Develop 3D DL model (*AppendiXNet*) to detect acute appendicitis	Video pretraining significantly improved the performance of DL model (accuracy from 57 to 72%, AUC from 0.72 to 0.81)
Sakai et al. [44]	2007	Japan	n 169 DL (**ANN**) vs ML (LR) Training: demographic, clinical and biochemical data	Diagnosis of acute appendicitis	ANN more accurate than LR (AUC 0.75 vs 0.72)

ML, Machine Learning; RF, Random Forest; DT, Decision Tree; K-NN, K-Nearest Neighbors; NB, Naïve Bayes; SVM, Support Vector Machine; GLM, Generalized Linear Model; DL, Deep Learning; ANN, Artificial Neural Network; LR, Logistic Regression; GBT, Gradient Boosted Tree; CART, Classification and Regression Tree; CNN, Convolutional Neural Network; AUC, Area Under the Curve

## AI in the prediction of acute appendicitis management

It is essential to state that the histopathological type of appendicitis directs the type of management [62]. In other words, predicting the kind of appendicitis can guide the choice of treatment. The appendicitis diagnosis could be, more simply, divided into only two subgroups: complicated appendicitis and uncomplicated appendicitis. This is because uncomplicated appendicitis can benefit from non-operative treatment. Diagnostic methods, including CT scans, are not very accurate in this distinction and they cannot be relied upon for defining when non-operative management can be used. In the study by Liang et al. [63] was developed a combined model with CatBoost based on selected clinical characteristics, CT visual features, deep learning features and radiomics features. They externally validated this combined model and compared it both with the DL radiomics model and the radiologist’s visual diagnosis through receiver operating characteristic curve analysis. In this context, a combined use of DL and radiomics model was effective in distinguishing complicated from uncomplicated forms, and therefore, in predicting patients who can benefit from non-operative management, with good accuracy.

There are three histopathological categories of acute appendicitis: simple appendicitis (SA), purulent appendicitis (PA) and gangrenous or perforated appendicitis (GPA). According to Kang et al. [64] peripheral blood biomarkers can recognize the pathological type of SA from PA and GPA. They collected the basic information and preoperative clinical and laboratory data of 146 patients diagnosed with acute appendicitis from the electronic medical records system, retrospectively. These included: age, gender, clinical sign and symptom scores, laboratory records: blood routine, coagulation, blood biochemistry, white blood cells, neutrophils, lymphocytes CD3+ T, CD4+ T, CD8+ T, CD19+ T, CD16+ 56+, natural killer, total T cell counts, helper T cell counts, inhibitors T, B cell counts, natural killer cell counts, CD4+/CD8+ ratio, C-reactive protein, procalcitonin and neutrophil to lymphocyte ratio. Two datasets involving SA and PA, or PA and GPA data, were organized, retrospectively. The two groups were named SA/PA and PA/GPA. Afterwards, ML logistic regression models were built. It showed that nausea and vomiting, abdominal pain time, neutrophils, CD4+ T cell, helper T cell, B lymphocyte, natural killer cell counts and CD4+/CD8+ ratio were predictive features for the SA/PA group. On the other hand, nausea and vomiting, abdominal pain time, the highest temperature, CD8+ T cell, procalcitonin and C-reactive protein were prevalent in the PA/GPA group. This information obtained thanks to AI can guide the therapeutic choice both regarding the surgical approach and the possible use of antibiotics.

Marcinkevics et al. [65] developed a user-friendly online appendicitis prediction tool for children with suspected appendicitis. In detail, they used ML techniques based on retrospective data from 430 children, to establish diagnosis, severity and management of appendicitis. The model could differentiate patients requiring primary surgery from those suitable for conservative management with or without antibiotics, identifying the characteristics determining a spontaneous regression of acute appendicitis. Although there is still few evidence in the literature, the aid of AI in identifying type and severity of appendicitis seems to guide the choice of treatment, avoiding surgery when not indicated (Table 3).

**Table 3:** A summary of the articles describing the role of artificial intelligence in the prediction of acute appendicitis treatment

Author	Year	Country	Method	Aim	Findings
Kang et al. [64]	2021	China	n 136 ML (**LR**) Training: demographic and clinical data, biomarkers	Predict pathological types of acute appendicitis	Peripheral blood biomarkers in addition with clinical features can predict SA from PA (AUC 0.93) and PA from GPA (AUC 0.85)
Liang et al. [63]	2023	China	n 1165 Catboost (**GBT**) Training: DL, Radiomics, clinical and CT features	Differentiate complicated from uncomplicated appendicitis	Combined DLR model best results (AUC 0.80)
Marcinkevics et al. [65]	2021	Switzerland	n 430 ML (**RF**, LR, GBM) Training: demographic and clinical data, biomarkers, US	Predict diagnosis, management and severity of appendicitis	RF best results (AUC of 0.94, 0.92, and 0.70, respectively, for diagnosis, management, and severity of appendicitis)

ML: machine learning, LR: logistic regression, SA: simple acute appendicitis, PA: purulent acute appendicitis, GPA: gangrenous or perforated acute appendicitis, GBT: gradient boosted tree, DL: deep learning, CT: computed tomography, DLR: deep learning radiomics, RF: random forest, GBM: generalized boosted regression model, US: ultrasonography, AUC: area under the curve

## AI in the prediction of postoperative complications of appendectomy

There are few data on the use of AI in investigating the outcome of patients undergoing appendectomy for acute appendicitis. The onset of intra-abdominal abscesses is the most frequent postoperative complication of such operations, especially in cases of complicated acute appendicitis [66]. The use of AI in relation to the occurrence of this complication has been poorly investigated. The most widespread studies in the literature focus on ML methods analyzing the surgical outcome of these patients. With the aid of RF techniques, after training with demographic, clinical and biomarker data, Eickhoff et al. [67] showed how the surgical outcome of patients with perforated appendicitis can be influenced by these factors. Outcomes such as the need for intensive postoperative treatment longer than 24 h and prolonged hospitalization longer than 7–15 days were predicted with high accuracy rates (88 and 76%, respectively).

Sepsis, a rare complication after appendectomy, was also investigated [68]. Various ML algorithms were used on a dataset of 223,214 appendectomy patients. LR, RF and GBT were the most accurate in predicting the occurrence of sepsis in these patients. After AI training with demographic, clinical and laboratory data, factors such as cardiac heart failure, exacerbation or diagnosis, acute renal failure and preoperative transfusion were significantly associated with the onset of postoperative sepsis. On the other hand, singular was the study by Ghomrawi et al. [69], in which a consumer-grade wearable device named *Fitbit* was used in the postoperative monitoring of appendectomy pediatric patients. This device was able to monitor parameters such as heart rate, physical activity and sleep pattern after discharge and associate them with the onset of postoperative symptoms or complications. These inputs, together with clinical and demographic data, allowed the development of ML models, specifically a balanced RF classifier capable of detecting with 83% of accuracy complicated appendicitis and with 70% of accuracy simple appendicitis, underlying such abnormal postoperative courses.

Only one study investigated ANN techniques in relation to postoperative complications. Indeed, according to a recent US study of 1574 patients, two ANNs with different architecture were able to predict post-appendicectomy abscess formation with high rates of accuracy, sensitivity and specificity, based on variables such as postoperative white blood cell count, intraoperative diagnosis, duration of surgery, antibiotic therapy completed, body temperature on the date of imaging and weight [70].

Hence, an increasing number of studies are focusing not only on the diagnosis of appendicitis but even on postoperative complications, to predict an abnormal course at an early stage and reduce the risks for the patient (Table 4).

**Table 4:** A summary of the articles describing the role of artificial intelligence in the prediction of acute appendicitis postoperative complications

Author	Year	Country	Method	Aim	Findings
Eickhoff et al. [67]	2022	Germany	n 163 ML (**RF**) Training: demographic, clinical and surgical data	Predict postoperative complications	Need for intensive care (accuracy 77%) Stay on ICU > 1 day (accuracy 88%) CD > 3 (accuracy 68%) Re-operation (accuracy 74%) SSI (accuracy 66%) AB-therapy (accuracy 79%) PO stay > 7 and 14 days (accuracy 76 and 84%)
Bunn et al. [68]	2021	USA	n 223,214 ML (**LR**, **RF**, **GBT**, SVM) Training: demographic, clinical and surgical data, biomarkers	Predict sepsis or septic shock	LR, RF and GBT best results (LR AUC 0.70, RF AUC 0.70, GBT AUC 0.70)
Ghomrawi et al. [69]	2023	USA	n 162 ML (**RF**) Training: PA, HR and sleep data recorded by *Fitbit*, demographic and clinical data	Predict abnormal recovery events in both complicated and simple appendicitis	RF accuracy in complicated appendicitis 83% RF accuracy in simple appendicitis 70%
Alramadhan et al. [70]	2022	USA	n 1574 DL (**ANN**) Training: demographic and clinical data, laboratory markers	Predict development of intra-abdominal abscess	ANN model 1 (sensitivity 70%, specificity 94%, accuracy 90%) ANN model 2 (sensitivity 82%, specificity 85%, accuracy 84%)

ML, machine learning; RF, random forest; LR, logistic regression; GBT, gradient boosted tree; SVM, support vector machine; DL, deep learning; ANN, artificial neural network; ICU, intensive care unit; CD, Clavien–Dindo score; SSI, surgical site infection; AB, antibiotic; PO, postoperative; AUC, area under the curve

## Conclusion

AI in surgery is not limited to ML, DL, natural language processing, and computer vision. The dream of autonomous actions in surgery is already here, albeit, in limited ways. Surgeons must understand the basics of AI and learn to better understand its potential benefits instead of insisting on resisting innovation. The use of AI really seems to be a valuable tool in helping patients suffering from acute appendicitis, not only in diagnosing the condition but also in guiding treatment, whether surgical or not, and in preventing postoperative complications. Unfortunately, its use is still not so frequent and its application in clinical practice limited. This is due to the fact that some changes should be made in medical regulation and insurance for allowing its diffusion. Moreover, clinicians should be trained to use it and liaise with digital experts. Its application will spread in the next years, probably. In our opinion, considering its cost and the needed training, it will involve the academic hospitals at first, mainly. Further studies are needed to understand which method is more effective than others regarding acute appendicitis, but the results seem promising so far.

## Data Availability

Not applicable.
